# Progress in Surgery

**Published:** 1897-03-06

**Authors:** 


### Progress in Surgery.

### DISEASES OF THE LARYNX, &c.

(*Continued from page 368.*)

**Leukæmic Infiltration.**—Two instances are reported. One, reported by Ebstein,[4] occurred in a female, aged seventy, with enlarged liver and spleen, and a large glandular swelling in the axilla, and mononuclear leucocytosis, with diminution of red corpuscles, had so much infiltration of the ventricular bands that the vocal cords could not be seen. The patient caught cold, and this was followed by rapid increase of the laryngeal infiltration, the glottis being reduced to a mere chink, requiring tracheotomy. After death leukæmic infiltration was found to involve the whole larynx. Mager[5] had a similar case in a man aged fifty-eight. The liver, spleen, glands in the neck, axillæ, and groins were enlarged, and leucocytosis was pronounced. He suddenly became hoarse, and had pain and irritation in the throat, and a fortnight later complained of dyspnœa. Laryngoscopic examination showed immobility of the right half of the larynx and swelling of the whole mucous membrane. The glottis rapidly became narrower, and lumpy swellings appeared on the hinder aspect of the right arytenoid cartilage. Schrötter now diagnosed leukæmic infiltration of the larynx. Tracheotomy was performed, but the wound became gangrenous, and the patient died in a few days. At the post-mortem examination, in addition to infiltration of the larynx with mononuclear leucocytes, perichondritis of the arytenoid and cricoid cartilages was found, which had led to œdema of the epiglottis and the aryepiglottidean fold. This perichondritis may have arisen from the rapid growth of bacilli from the mouth in the favourable nidus resulting from the leukæmic disease.

**Varicella.**—Marfan and Halle[6] report two cases of varicella of the larynx. (1) A boy, three years of age, was suddenly seized with great dyspnœa and paroxysms of suffocation, and tracheotomy was performed. The case was suspected to be diphtheria, and after injections of anti-toxin had been given, a typical eruption appeared on the body. Breathing soon became normal, and the patient recovered. (2) A child, aged nine months, with typical eruption extensively over the body, had marked stridor and dyspnœa. Broncho-pneumonia developed, and the patient soon died. At the post-mortem examination shallow ulceration was found, about the size of a pea, on the posterior part of the right vocal cord, probably a ruptured vesicle.

**Membranous Sore Throat.**—A case of pelliculous sore throat is recorded by Gougenheim.[7] A man, about forty-five years of age, suddenly developed violent dyspnœa. When the dyspnœa had quieted, and the throat was very easily examined, the left side of the soft palate and the left tonsil were found to be covered with an extensive, continuous greyish-white exudation, presenting the aspect of a true diphtheritic false membrane. The rest of the throat was very red. Laryngoscopic inspection revealed a certain quantity of muco-purulent liquid, but the mucous membrane, slightly tumefied and injected, did not present any false membrane on its surface. The bacteriological examination of false membrane removed from the soft palate and the tonsil, and of the liquids in the larynx, failed to reveal the bacillus of diphtheria in any of the cultures made from these various regions. Nothing was encountered but the chains of streptococci, absolutely pure and without admixture.

The dyspnœa soon disappeared completely and the voice became normal. Nevertheless, the patient did not improve, and the fever continued constant. Serious complications ensued in connection with the pleura, the left lung, and the articulation of the left clavicle with the adjoining bone. At the same time the urine revealed the presence of a notable quantity of albumen.

At the moment when the patient seemed entering upon convalescence, the fever and the albumen disappearing little by little, he was suddenly carried off in instant by cardiac symptoms, which the autopsy showed to be due to a purulent pericarditis, the existence of which had not been suspected during the life of the patient. In addition to this pericarditis, the autopsy revealed pulmonary congestion, from apparent vestiges of the purulent pleura of the left side, and of pus in the articulations of the left clavical.

Another pseudo-diphtheritic case is recorded by Glover,[8] in which the pharynx, nose, and larynx were involved.

The case occurred in a woman, sixty-seven years of age, the subject of a chronic bronchitis with subacute exacerbations every spring. On February 13th, 1896, she was taken with slight fever with little chills, loss of appetite, followed on the next day by a coryza soon accompanied with bronchitis. Her temperature became elevated every evening, the cough became very intense and persistent, with very liquid aud abundant whitish expectoration. On the 17th hoarseness began in the morning, increasing toward evening to complete aphonia, fever became more marked and permanent, and the respiration difficult. The throat did not present any exudation. On February 18th she was admitted to the Hospital Lariboisiere very much excited by increasing impediment of respiration. The vestibule of the larynx was filled with frothy mucus, and as this was coughed out appeared to be covered with thick exudation, disseminated in points and in large plaques on the laryngeal face of the epiglottis, on the ventricular bands, and on both vocal bands. The laryngeal isthmus presented the aspect of a greyish infundibulum of very irregular surface, formed by the croupous layer of pseudo-membrane. Islets of exudation, easily removable, existed on the base of the tongue posteriorly, and on the sides of the lingual tonsil; the subjacent mucous membrane bled in the neighbourhood of each attachment. The aspect of the buccal pharynx presented the appearance of a very extended exudation, such as appears in an undoubted diphtheria.

Rhinoscopy, both anterior and posterior, gave no appreciable results. The dyspnœa was a little peculiar. Slight suction at the suprasternal fossa, and movements of the wings of the nose; laborious respiration with active participation of the thoracic muscles; feeble depression at the epigastric region synchronous with the commencement of the inspiration; inspiratory laryngeal stridor existing for a portion of the phase of inspiration, the expiration being made almost without noise.

Despite persistent difficulty in respiration, tracheotomy was not performed because the multiplicity of obstacles to the respiration seemed to implicate the lungs more than the larynx.

Although the repeated bacteriological examination disclosed nothing but staphylococci, the temperature became elevated, urine diminished, showing albumen. The patient died at midnight.

The post-mortem showed that the pseudo-membranes extended from the posterior nasal outlets down to the third divisions of the bronchi; while the only bacteriological element was the staphylococci.

[4] Wien. Klin. Woch, May 18, 1896; Epit. B.M.J., Oct. 31, 1896. [5] Wien. Klin. Woch, June 25, 1896; Epit. B.M.J., Oct. 31, 1896. [6] Rev. Mens. des Mal. de l'enfance, Jan., 1896; Med. News, Sept, 5, 1896. [7] Annales des Mal. de l'oreille du Lar., etc., Oct., 1896; Americ. Journ. of Med. Sc., Dec., 1896. [8] Annales des Mal. de l'oreille du Larynx, Nov., 1896; Amer. Journ. of Med. Sc., Nov., 1896.

### GENITO-URINARY SURGERY.

**The Surgery of the Prostate.**—In the "Progress in Surgery" in The Hospital for November 16th, 1895, reference was made to Dr. White's 111 cases of castration for hypertrophy of the prostate. Dr. Cabot, of Boston, has collected the records of 99 additional cases, and contributes a very valuable paper[1] on the subject, which, together with a paper[2] by Dr. William White, gives a very good idea of the present position of the operation. Dr. Cabot has not only utilised published cases, but wrote to a large number of American Surgeons for details of their unpublished cases, so that he might obtain a fairly correct idea of the mortality of the operation. Out of Cabot's 99 cases 20 died; and out of the 104 cases collected by White 19 died, which gives a mortality of 19·4 per cent. in the 203 cases. But Dr. White, recognising the fact that many of the patients who submit to castration are already suffering from diseases of the bladder and kidneys, which frequently prove fatal, considered that 13 out of his fatal cases could be eliminated when considering the true mortality of the operation. Dr. Cabot objects to this, and says, "Such a revision of statistics is proper when we are comparing them with other operations done upon patients in good health, but it is manifestly improper when we are making comparison of results with other operations carried out upon the same class of patients"; but Dr. White argues that "the ease and celerity with which castration can be performed have led to its adoption in many cases of disease at a stage, both local and general, which would have been regarded as a positive contra indication to any other operation," and maintains that it is "proper to exclude from the estimates of mortality due to the operation many cases that were manifestly practically moribund when the testicles were removed"; and he considers that ten out of Dr. Cabot's 20 fatal cases certainly ought to be excluded, because the patients were evidently too ill to bear any operation, or because they died from causes quite unconnected with it, or in spite of it, from renal disease, secondary to long-standing prostatic obstruction. Very few patients seem to have died from the operation, as we should expect, but, unfortunately, it is often resorted to so late in the case, and, therefore, in such a desperate condition of the patient, that they die in spite of an operation the good effects of which are only gradually produced. A table of 92 cases recently collected for Dr. White by Dr. Wood, of Philadelphia, shows nine deaths, or a mortality of 9·78 per cent. Dr. White thinks this reduction of mortality is probably due to a better selection of cases, and is likely to continue, and that it confirms the view which he originally expressed that the operation *per se* ought not to have a mortality of more than about 7 per cent. All fatal cases have been included in this list, but Dr. White shows that two might well be eliminated, which would reduce the mortality to 6·5. How does the mortality of castration compare with that of prostatectomy? Cabot considers, from a study of several tables of cases of prostatectomy, that the death-rate is about the same with both operations; but Dr. White points out that the mortality of prostatectomy is high in proportion to the age of the patient, and is on this account considered by some surgeons with considerable experience of the operation contra-indicated after 65, and he also recalls the fact that castration is resorted to in cases so desperate that prostatectomy would not be thought of.

Dr. Cabot also considers the mental effects of castration. In all the reported cases which he has studied he has never found any in which there has been a loss of masculine characteristics, or any appearance of feminine characteristics, after castration performed in middle or advanced age. In his table of 99 cases there were 11 instances of mental disturbance immediately following castration—in six a serious maniacal condition, and in five others a considerable loss of mental balance. In one case of mania injections of testiculin were used, apparently with considerable benefit. Dr. Cabot suggests that a gradual failure of vital power, without any marked change in the symptoms, leading to a fatal result after castration, may be due to removal of testicular secretion, which, as Brown-Séquard believed, may help to maintain the vital force of the patient.

Restoration of function after castration has been very encouraging. Cabot concludes from the study of the cases, that we can say to our inquiring patients "You have eight chances in ten of getting through this operation all right, and if you are successful in this you have again eight chances in ten of getting very substantial relief from your urinary troubles." Dr. White would give (as we have already seen) the mortality as one in ten. In 20 cases of long-standing retention this condition disappeared after castration, and in one case after existing for 11 years and in another for 18 years. Twenty-four cases in which the power of micturition had not been wholly lost but was greatly impaired, making the act frequent and often painful, were greatly relieved by castration, and the amount of residual urine diminished.

[1] Annals of Surgery, Sept., 1896. [2] Ibid.

